# Gender-based differences in interaction effects between childhood maltreatment and problematic mobile phone use on college students’ depression and anxiety symptoms

**DOI:** 10.1186/s12888-023-04777-x

**Published:** 2023-04-25

**Authors:** Yulin Zhang, Shuqin Li, Huiqiong Xu, Zhengge Jin, Ruoyu Li, Yi Zhang, Yuhui Wan

**Affiliations:** 1 The Second Clinical Medical College, Anhui Medical University, No. 81 Meishan Road, Hefei, Anhui 230032 China; 2 Department of Maternal, Child and Adolescent Health, School of Public Health, Anhui Medical University, No. 81 Meishan Road, Hefei, Anhui 230032 China; 3 NHC Key Laboratory of Study on Abnormal Gametes and Reproductive Tract, No. 81 Meishan Road, Hefei, Anhui 230032 China; 4 MOE Key Laboratory of Population Health Across the Life Cycle, No. 81 Meishan Road, Hefei, Anhui 230032 China; 5 Department of Endocrinology, The First Affiliated Hospital of Anhui Medical University, Hefei, Anhui 230022 China

**Keywords:** Depression, Anxiety, Problematic mobile phone use, Childhood maltreatment, Interaction, Gender, College students

## Abstract

**Background:**

Childhood maltreatment and problematic mobile phone use are risk factors for depression and anxiety symptoms among college students. However, how the interaction between the two factors affects depression and anxiety has yet to be validated. This study aimed to investigate the independent and interaction effects of childhood maltreatment and problematic mobile phone use on depression and anxiety among college students and explored gender-based differences in these associations.

**Method:**

A cross-sectional study was conducted from October to December 2019. We collected data from 7623 students at two colleges in Hefei and Anqing cities in Anhui Province, China. Multinomial logistic regression models were performed to explore the associations of childhood maltreatment and problematic mobile phone use with depression and anxiety symptoms and their interaction effects on depression and anxiety symptoms.

**Results:**

Childhood maltreatment and problematic mobile phone use were significantly associated with increased risk of depression and anxiety symptoms (P < 0.001). Moreover, following adjustments for covariates, there was a multiplicative interaction between childhood maltreatment and problematic mobile phone use on depression and anxiety symptoms (P < 0.001). Gender-based differences were also observed in the associations. For instance, depression was more common in males and male students with childhood maltreatment were at higher risk of depression-only symptoms.

**Conclusion:**

Focusing on childhood maltreatment and problematic mobile phone use could facilitate a reduction in the occurrence of depression and anxiety symptoms in college students. Furthermore, it is necessary to develop gender-targeted intervention strategies.

**Supplementary Information:**

The online version contains supplementary material available at 10.1186/s12888-023-04777-x.

## Background

College students are particularly vulnerable to depression and anxiety because during this developmental stage many changes occur, such as having to form new interpersonal relationships and adjusting to new academic stresses [1]. The overall prevalence of depression symptoms during the college years in participants in 113 studies was 28.4% (95% confidence interval [CI]: 25.7-31.2%) [2]. College students with depression may show maladaptive coping behaviors such as suicidal behaviors [3]. According to Li et al.’s (2021) meta-analysis, 19% of the global population self-reports experiencing anxiety [4]. People with anxiety report a lower quality of life than those with low anxiety levels [5]. Some studies have shown that psychiatric problems in childhood tend to manifest as a single disorder, while the crossover and interaction between psychiatric pathogenesis increases in adolescence, with more pronounced changes in co-morbid symptoms during this period [6].

Co-morbidity of depression and anxiety is common [7]. Data from the American Co-morbidity Survey showed that 51.2% of depressed patients had a lifetime co-morbid anxiety disorder, with a 23.7% co-morbidity prevalence in the past 12 months [8]. National surveys show that 69% of depressed patients also have a co-morbid anxiety disorder [9]. Compared with single psychiatric disorders, patients with co-morbid depression and anxiety are characterized by severe symptom and functional impairment, long duration of illness, high suicide rate, and poor prognosis [10]. This makes co-morbidity of depression and anxiety one of the hot issues in public health and its related fields. Therefore, the increasing emphasis on the factors that cause depression and anxiety and the association between these factors is attracting considerable attention.

Childhood maltreatment is defined as abuse and neglect of people under the age of 18 years. It may include any acts of commission or omission by a parent or other caregiver that results in actual or potential harm to a child, and can occur in a home, at school, or in the communities the child interacts with [11]. Childhood maltreatment is usually divided into two subcategories: acts of omission (emotional and physical neglect) and acts of commission (physical, emotional, and sexual abuse) [12]. In a meta-analysis of 32 studies, the pooled prevalence of childhood maltreatment among college students in China was 64.7% (95% CI: 52.3-75.6%) [13]. Exposure to childhood maltreatment may further increase severe mental health problems, including depression and anxiety symptoms, in early adulthood [14–16]. One study suggests that if childhood maltreatment were reduced by 10–25%, between 31.4 million and 80.3 million cases of depression and anxiety could potentially be prevented worldwide [17].

Today’s college students have grown up in parallel with the rapid development of digital technologies, such as smartphones, which have become an integral part of their daily lives [18]. Almost all college students use their mobile phones to access entertainment and social connections. Problematic mobile phone use is defined as inappropriate or excessive use of mobile phones, leading to physiological and psychological dysfunction [19]. For example, in Anhui, China, the prevalence of smartphone addiction among studied college students was 29.8% [20]. In a survey of 2000 university students, 85% agreed that they regularly checked their smartphones to determine the time, and 75% responded that they kept their smartphones with them when they went to bed. These figures suggest that excessive smartphone use can be problematic [21]. Recent evidence found that problematic mobile phone use may be a risk factor for depression and anxiety in college students [22–24].

A history of childhood maltreatment or problematic mobile phone use is common in college students, and adversely affects their mental health in the short and long term. Notably, the available findings suggest that childhood maltreatment positively predicts subsequent problematic mobile phone use. That is, childhood maltreatment as an early and distal risk factor increases the risk of problematic mobile phone use [25]. As an inadequate coping strategy, problematic mobile phone use may exacerbate the consequences of childhood maltreatment. However, the interaction effects between childhood maltreatment and problematic mobile phone use on college students’ depression and anxiety symptoms have not been researched, despite childhood maltreatment and problematic mobile phone use being highly correlated [26, 27]. The current study hypothesized that childhood maltreatment and problematic mobile phone use are associated with depression and anxiety symptoms and have a synergistic effect among college students.

Some studies suggest that gender-based differences are robust in both childhood maltreatment [28] and problematic mobile phone use [29]. For instance, according to data from the United States and Germany [30, 31], female students are more likely to endure neglect, and emotional and sexual abuse, while boys are more likely to experience physical abuse. Jiang et al. [32] found that women were more likely to rely on mobile phones compared to men. Gender-based differences have also been observed in the prevalence of depression and anxiety symptoms [33]. A meta-analysis found that the prevalence of depression symptoms among college students in China from 1997 to 2015 ranged from 3.0 to 80.6%, with a pooled prevalence of 23.8%, of which the prevalence of depression symptoms among male students was 30.0%, slightly higher than the prevalence of depression symptoms among female students (27.1%) [2]. The extant literature on gender-based differences in the associations of childhood maltreatment and problematic mobile phone use on depression and anxiety symptoms is limited. Documenting potential gender-based differences is important because it may contribute to the development of gender-specific interventions for depression and anxiety symptoms.

Therefore, our study first aimed to investigate the independent effects of childhood maltreatment and problematic mobile phone use on depression and anxiety symptoms in college students. Second, we aimed to examine the interaction effects between childhood maltreatment and problematic mobile phone use on depression and anxiety symptoms. Third, we sought to find any apparent gender-based differences in these associations.

## Method

### Aim and Design of the study

This study aimed to investigate the independent and interaction effects of childhood maltreatment and problematic mobile phone use on depression and anxiety among college students and explored gender-based differences in these associations. We conducted a cross-sectional survey from October to December 2019 at two colleges in Hefei and Anqing City in Anhui Province, China.

### Participants

A total of 8198 students with a mean age of 19.66 years (SD 1.11) were recruited. Participants from both colleges were either freshmen or sophomores. Participation was completely voluntary and anonymous. In this study, one class session was organized with the assistance of well-trained investigators for students to complete self-report questionnaires in classrooms. The study purpose was explained at the beginning of the survey, and the principles of confidentiality were emphasized. Participants who had more than 5% of items with missing data were excluded. Thus, data from 7623 (93.0%) were analyzed. More female, compared to male, students participated in this survey (74.1% vs. 25.9%, respectively). The study design and data collection procedures were both approved by the Ethics Committee of Anhui Medical University (20,170,290). Informed consent was obtained from all students.

### Measures

In this study, participants completed questionnaires designed to measure sociodemographic indicators, childhood maltreatment, problematic mobile phone use, and the prevalence of depression and anxiety symptoms. Considering the problem of common method bias due to self-reporting, Harman’s single factor test was conducted and the results showed that 15 factors had eigenvalues greater than 1 and the variance explained by the first factor was 18.47% (less than the 40% threshold), indicating no serious problem of common method bias.

#### Demographic information

Information regarding gender, grade, residential area, only child or not, perceived family economic status, and the number of close friends was collected. Residential areas were classified into two categories: urban and rural. Only child or not was divided into two categories: yes and no. Perceived family economic status was categorized as poor, moderate, or good. The number of close friends included four categories: 0, 1–2, 3–5, and ≥ 6.

#### Childhood maltreatment

Childhood maltreatment was evaluated with the Childhood Trauma Questionnaire-Short Form (CTQ-SF) [34]. The Chinese version of the CTQ-SF has demonstrated good reliability and validity among Chinese college students [35]. The questionnaire included 28 items (25 clinical items and 3 validity items), including five dimensions: emotional abuse (EA), physical abuse (PA), sexual abuse (SA), emotional neglect (EN), and physical neglect (PN). Items were rated on a five-point scale: 1 = never, 2 = rarely, 3 = sometimes, 4 = often, and 5 = very often. The scores of each of the five subscales of the CTQ-SF range from 5 to 25. Based on ROC curve estimation of sensitivity and specificity for each subscale against criteria measures from Evaluation of Life-time Stressors, Bernstein and Fink proposed cut-offs to create categories of severity: None or minimal (EA: 5–8; PA: 5–7; SA: 5; EN: 5–9; PN: 5–7); Low (EA: 9–12; PA: 8–9; SA: 6–7; EN: 10–14; PN: 8–9); Moderate (EA: 13–15; PA: 10–12; SA: 8–12; EN: 15–17; PN: 10–12); and Severe (EA: ≥ 16; PA: ≥ 13; SA: ≥ 13; EN: ≥ 18; PN: ≥ 13) [12]. In this study, participants with scores higher than any of the following subscale thresholds were considered to have experienced childhood maltreatment: EA ≥ 9; PA ≥ 8; SA ≥ 6; EN ≥ 10; and PN ≥ 8 [36]. In this study, Cronbach’s α coefficient was 0.793.

#### Problematic mobile phone use

Problematic mobile phone use was evaluated by the Self-Rating Questionnaire for Adolescent Problematic Mobile Phone Use (SQAPMPU) [37], which includes three dimensions: withdrawal symptoms, craving, and physical and mental health statuses. The SQAPMPU includes 13 items (e.g., “I play on my phone too much, resulting in lack of sleep,” “I become irritable if I have to turn off my phone for a meeting, dinner event, or during a movie,” “I feel like I need to spend more time on my phone to be satisfied”). Items were rated on a five-point scale: 1 = not at all, 2 = slightly, 3 = moderately, 4 = strongly, and 5 = extremely. The total scores ranged from 13 to 65. Using the 75th percentile as the cutoff point [38], problematic mobile phone use behaviors were categorized as “No” (< 26) or “Yes” (≥ 26). Cronbach’s α coefficient of the scale in the current study was 0.912.

#### Depression and anxiety symptoms

The Self-rating Depression Scale (SDS) was used to measure depression severity [39]. The Self-Rating Anxiety Scale (SAS) was used to measure anxiety severity [40]. Each scale contains 20 items rated on a four-point scale: 1 = no or a little of the time, 2 = some of the time, 3 = a good part of the time, and 4 = most of the time or all the time. Zung chose to convert raw scores (range 20–80) into index scores (range 25–100) by dividing the sum of the raw scores by 80, and multiplying by 100 with clinical cut-offs being given in terms of the latter [41]. The cutoff score of the SDS was 53 (index scores ≥ 53 indicate depression) and the cutoff score of the SAS was 50 (index scores ≥ 50 indicate anxiety) [42]. In this study, Cronbach’s α coefficients were 0.904 for SDS and 0.811 for SAS.

### Analysis

Descriptive statistics were applied to describe the students’ demographic information, childhood maltreatment, problematic mobile phone use, and their depression and anxiety symptoms. A categorical variable was presented as frequency (n) and percentage (%), and the differences between the groups were compared using chi-square tests. We examined the interaction effects of childhood maltreatment and problematic mobile phone use on depression and anxiety symptoms by evaluating the multiplicative and additive interactions. The multiplicative interactions were used to assess whether the risk due to having both factors is greater than the product of the risks due to each factor alone. The additive interactions were used to assess whether the risk due to having both factors is greater than the sum of the risks due to each factor alone. Multinomial logistic regression models were used in which individuals were categorized into four groups: Depression only, Anxiety only, Depression and Anxiety, or No Depression or Anxiety.

#### Main analysis

We established four logistic regression models using self-rated depression and anxiety symptoms as dependent variables. **Model 1**: We estimate the odds ratio (OR) and 95% CI of the independent effects of childhood maltreatment and problematic mobile phone use on depression and anxiety, after adjusting for other confounding factors. **Model 2**: To explore the multiplicative interaction effects, we set four dummy variables based on whether they were exposed to childhood maltreatment and problematic mobile phone use, and no childhood maltreatment with no problematic mobile phone use was used as a reference group. **Model 3**: To investigate whether gender moderates the association between childhood maltreatment or problematic mobile phone use on depression and anxiety symptoms, we set four dummy variables based on whether participants were exposed to childhood maltreatment or problematic mobile phone use and whether participants were female or male, and female with yes childhood maltreatment or yes problematic mobile phone use was used as a reference group. **Model 4**: We looked at the additive interaction of childhood maltreatment and problematic mobile phone use on co-morbidity of depression and anxiety among college students. Co-morbidity of depression and anxiety was coded as 1 and the rest as 0. In the additive model, we derived the relative excess risk due to interaction (RERI), attributable proportion due to interaction (AP), and synergy index (SI) [43, 44]. An Excel table created by Andersson [45] was applied to calculate the RERI, AP, and SI, with a 95% CI. If the 95% CI of RERI and AP range did not include 0, and the corresponding SI range did not include 1, then the additive interactions were judged as statistically significant.

Multinominal logistic regression was performed in all models except for model 4. Statistical analysis was performed using IBM SPSS 23.0, and statistical significance was set at P < 0.05.

#### Sensitivity analysis

We also carried out a sensitivity analysis. We conducted a one-way analysis of variance (ANOVA) on the basis of all variables being continuous variables.

## Results

### Characteristics of the participants

Among 7623 participants, 74.1% (n = 5652) were female students. The prevalence of depression, anxiety, and co-morbidity of depression and anxiety symptoms were 20.0%, 20.3%, and 10.1%, respectively. Childhood maltreatment and problematic mobile phone use were observed in 70.2% and 26.1% of participants, respectively. The prevalence of childhood maltreatment was higher among male than female students (P < 0.05), with differences in depression and anxiety symptoms by gender (P < 0.05). However, there were no statistical differences in the prevalence of problematic mobile phone use among college students of different genders (P > 0.05). The differences in the prevalence of depression only, anxiety only, depression and anxiety, and no depression or anxiety were statistically significant across gender, only child or not, perceived family economic status, and number of close friends (all P values < 0.05). Table 1 presents the Chi-square test results and significant differences in the selected characteristics based on depression and anxiety symptoms are. In addition, more descriptive information, for instance on frequencies and percentages for each variable (Table S1) and the means, ranges, and correlations of the subscales (Table S2), has been included in the “Supplemental Material.”

**Table 1:** Comparison of the prevalence of depression and anxiety symptoms among different groups of college students

Variables	Depression and Anxiety (%)	Depression Only (%)	Anxiety Only (%)	No Depression or Anxiety (%)	*χ*^2^-value	*P*-value
Grade					3.157	0.368
freshman	424 (10.1)	432 (10.3)	443 (10.5)	2912 (69.2)		
sophomore	347 (10.2)	318 (9.3)	336 (9.8)	2411 (70.7)		
**Gender**					**17.353**	**0.001**
male	222 (11.3)	233 (11.8)	194 (9.8)	1322 (67.1)		
female	549 (9.7)	517 (9.1)	585 (10.4)	4001 (70.8)		
Residential area					0.701	0.873
rural	622 (10.1)	600 (9.7)	633 (10.2)	4322 (70.0)		
urban	149 (10.3)	150 (10.4)	146 (10.1)	1001 (69.2)		
**Only child or not**					**8.440**	**0.038**
yes	168 (10.3)	191 (11.7)	158 (9.7)	1119 (68.4)		
no	603 (10.1)	559 (9.3)	621 (10.4)	4204 (70.2)		
**Perceived family economic status**					**99.621**	**< 0.001**
poor	372 (13.3)	346 (12.4)	292 (10.5)	1779 (63.8)		
moderate	375 (8.2)	384 (8.4)	457 (10.0)	3367 (73.5)		
good	24 (9.6)	20 (8.0)	30 (12.0)	177 (70.5)		
**Number of close friends**					**267.107**	**< 0.001**
0	56 (27.6)	47 (23.2)	13 (6.4)	87 (42.9)		
1–2	382 (13.4)	336 (11.8)	300 (10.5)	1829 (64.2)		
3–5	265 (7.9)	302 (9.0)	350 (10.4)	2433 (72.6)		
≥ 6	68 (5.6)	65 (5.3)	116 (9.5)	974 (79.6)		
**CM**					**321.163**	**< 0.001**
yes	700 (13.1)	639 (11.9)	573 (10.7)	3438 (64.3)		
no	71 (3.1)	111 (4.9)	206 (9.1)	1885 (82.9)		
**PMPU**					**1012.182**	**< 0.001**
yes	486 (24.4)	232 (11.7)	382 (19.2)	888 (44.7)		
no	285 (5.1)	518 (9.2)	397 (7.0)	4435 (78.7)		
CM*PMPU					**1328.843**	**< 0.001**
no*no	34 (1.8)	74 (4.0)	103 (5.6)	5323 (69.8)		
yes*yes	449 (28.9)	195 (12.5)	279 (17.9)	633 (40.7)		
no*yes	37 (8.6)	37 (8.6)	103 (23.8)	255 (59.0)		
yes*no	251 (6.6)	444 (11.7)	294 (7.7)	2805 (73.9)		

CM: Childhood maltreatment

PMPU: Problematic mobile phone use

### Independent effects of childhood maltreatment and problematic mobile phone use on depression and anxiety symptoms (Model 1)

As shown in Table 2, after adjusting grade, gender, residential area, only child or not, perceived family economic status, and number of close friends for the college students, childhood maltreatment and problematic mobile phone use remained significantly associated with an increased likelihood of depression only, anxiety only, and co-morbidity of depression and anxiety symptoms. After gender stratification, no significant inconsistencies were observed in the results (all P values < 0.05).

**Table 2:** Multinominal logistic regression of associations of CM and PMPU on depression and anxiety

Variables	Depression only^a^				Anxiety only^a^				Depression and Anxiety^a^		
	OR	95% CI	*P*-value		OR	95% CI	*P*-value		OR	95% CI	*P*-value
**CM** ^*^											
no	1.00				1.00						
yes	2.845	2.303–3.516	< 0.001		1.496	1.262–1.774	< 0.001		4.823	3.747–6.208	< 0.001
**PMPU** ^*^											
no	1.00				1.00						
yes	2.163	1.820–2.571	< 0.001		4.780	4.081–5.598	< 0.001		8.210	6.959–9.685	< 0.001
CM*PMPU ^**#**^											
no*no	1.00				1.00				1.00		
yes*yes	5.990	4.502–7.971	< 0.001		6.853	5.362–8.758	< 0.001		29.779	20.724–42.789	< 0.001
no*yes	3.081	2.028–4.683	< 0.001		6.361	4.695–8.617	< 0.001		6.700	4.120-10.897	< 0.001
yes*no	3.143	2.434–4.059	< 0.001		1.634	1.294–2.065	< 0.001		3.846	2.670–5.541	< 0.001

^a^: Adjusting for grade, gender, residential area, only child or not, perceived family economic status, number of close friends

CM: Childhood maltreatment

PMPU: Problematic mobile phone use

^*^: Model 1

^**#**^: Model 2

### Multiplicative interactions between childhood maltreatment and problematic mobile phone use on depression and anxiety symptoms (Model 2)

Table 2 shows the multiplicative interaction between childhood maltreatment and problematic mobile phone use on depression and anxiety symptoms. A full review of the multinominal logistic regression model including all covariates is already included in the supplemental material (Tables S3 - S5). Among the students, 3794 (49.8%) suffered childhood maltreatment without problematic mobile phone use and 432 (5.7%) had only problematic mobile phone use without childhood maltreatment. Students who suffered childhood maltreatment and problematic mobile phone use were 1556 (20.4%). Compared with those without childhood maltreatment or problematic mobile phone use, college students who suffered childhood maltreatment and problematic mobile phone use had higher adjusted ORs of depression only, anxiety only, and co-morbidity of depression and anxiety symptoms (depression only: OR = 5.990, 95% CI = 4.502–7.971, P < 0.001; anxiety only: OR = 6.853, 95% CI = 5.362–8.758, P < 0.001; depression and anxiety: OR = 29.779, 95% CI = 20.724–42.789, P < 0.001). This indicates multiplicative interactions between childhood maltreatment and problematic mobile phone use after adjusting for other confounding factors.

### Multiplicative interactions between childhood maltreatment or problematic mobile phone use and gender on depression and anxiety symptoms (Model 3)

Table 3 shows the effects of gender on depression and anxiety symptoms among college students with childhood maltreatment or problematic mobile phone use. Among college students who experienced childhood maltreatment, male students were at higher risk of depression-only symptoms compared to their female counterparts (OR: 1.300, 95% CI: 1.075–1.573, P < 0.05). Among college students with problematic mobile phone use, there was no statistical difference in the risk of depression and anxiety symptoms between male and female students.

**Table 3:** Multinominal logistic regression of interactions between CM or PMPU and gender on depression and anxiety

Variables	Depression only^a^				Anxiety only^a^				Depression and Anxiety^a^		
	OR	95% CI	*P*-value		OR	95% CI	*P*-value		OR	95% CI	*P*-value
CM*gender											
yes*female	1.00				1.00				1.00		
no*male	0.418	0.271–0.644	< 0.001		0.672	0.469–0.961	0.030		0.246	0.143–0.424	< 0.001
yes*male	1.300	1.075–1.573	**0.007**		1.005	0.815–1.239	0.965		1.181	0.968–1.440	0.101
no*female	0.376	0.295–0.478	< 0.001		0.739	0.607–0.899	0.003		0.237	0.177–0.317	< 0.001
PMPU*gender											
yes*female	1.00				1.00				1.00		
no*male	0.613	0.480–0.782	< 0.001		0.204	0.157–0.263	< 0.001		0.138	0.104–0.182	< 0.001
yes*male	1.096	0.786–1.529	0.587		1.053	0.798–1.389	0.717		1.280	0.988–1.659	0.062
no*female	0.450	0.367–0.553	< 0.001		0.220	0.183–0.264	< 0.001		0.137	0.113–0.167	< 0.001

a: Adjusting for grade, gender, residential area, only child or not, perceived family economic status, number of close friends

CM: Childhood maltreatment

PMPU: Problematic mobile phone use

### Additive interactions of childhood maltreatment and problematic mobile phone use on co-morbidity of depression and anxiety among college students (Model 4)

Synergistic effects of childhood maltreatment and problematic mobile phone use on co-morbidity of depression and anxiety among college students are observed (Tables S6, S7). The RERI, AP and SI values were 11.675 (6.913–16.438), 0.620 (0.516–0.724), and 2.900 (2.106–3.993), respectively.

### Sensitivity analysis

We found that the main and interaction effects of childhood maltreatment and problematic mobile phone use on depression and anxiety symptoms among the college students remained statistically significant (Table S8).

## Discussion

Our study found that the prevalence of depression (20.0%) and anxiety (20.3%) among college students was lower than that reported in previous studies [46, 47]. The prevalence of c^o−morbidity of depression and anxiety^ among participants in this study was 10.1%, which was lower than the prevalence of c^o−morbidity of depression and anxiety^ among medical students in Ethiopia (20.0%) [48] and university students in mainland China during the COVID-19 pandemic (20.9%) [49], and consistent with the findings of a survey of US university students (10.0%) [50]. The inconsistent outcomes may be related to the different survey periods, different psychological scales used and the existing socio-cultural differences among the countries. A descriptive analysis of gender-based differences in depression and anxiety symptoms among college students showed differences in depression and anxiety symptoms by gender. For instance, depression was more common in males. Regarding gender-based differences, there are no consistent conclusions in the literature [51, 52]. The inconsistent outcomes may be related to the different sampling methods and the proportion of male and female students in these studies.

Previous reports have shown that childhood maltreatment is significantly associated with depression and anxiety symptoms [53, 54]. Childhood maltreatment can cause deficits or difficulties in understanding and regulating emotions, which may mediate the relationship between childhood maltreatment and depression and anxiety symptoms [55]. Furthermore, problematic mobile phone use is a risk factor that affects Chinese students’ mental health [56, 57]. Problematic mobile phone use can disrupt the normal sleep patterns of college students, which leads to more depression and anxiety symptoms [58]. Our study confirmed that childhood maltreatment and problematic mobile phone use predicted depression and anxiety symptoms, consistent with previous studies, even after full adjustment for potential confounding factors.

Although childhood maltreatment and problematic mobile phone use are well-known risk factors for depression and anxiety symptoms in college students, little is known about the interactions between childhood maltreatment and problematic mobile phone use, or about how childhood maltreatment and problematic mobile phone use jointly influence depression and anxiety. Our study found a synergistic effect between childhood maltreatment and problematic mobile phone use on depression and anxiety symptoms. College students with childhood maltreatment and problematic mobile phone use are at a significantly increased risk of depression and anxiety. This was confirmed in the interaction analyses in the study, indicating that we should pay attention to childhood maltreatment and problematic mobile phone use to reduce the incidence of depression and anxiety symptoms. In addition, a history of childhood maltreatment can frustrate young people’s basic psychological needs and reduce their self-compassion, which increases the likelihood of problematic mobile phone use [25]. Therefore, childhood maltreatment is considered a risk factor for problematic mobile phone use among college students [59]. The presence of problematic mobile phone use, in turn, may further increase the impact of childhood maltreatment on depression and anxiety symptoms. Furthermore, as mobile phone use becomes prevalent, we might hypothesize that children with problematic mobile phone use are also more likely to experience childhood maltreatment and, similarly, that the experience of childhood maltreatment increases the risk of depression and anxiety symptoms in college students with problematic mobile phone use. The serious and long-term consequences of childhood maltreatment and problematic mobile phone use warrant increased investment in prevention and therapy from early childhood.

Male students with a history of childhood maltreatment were at higher risk of depression-only symptoms compared to their female counterparts, which accords with the claim that being female appeared to be protective against depression symptoms in college educated people [60]. Hence, the greater social interconnectedness and preferential cooperativeness associated with femininity may be more important than the individualistic traits associated with masculinity as a protective factor against psychological distress in contemporary society [60]. Alternatively, this may be related to the fact that female students outperform their male counterparts and adapt more easily to the pressure of the curriculum. Our findings are inconsistent with those of Powers et al. [16] who concluded that females with a history of childhood maltreatment had higher levels of depression. The results and interpretation of gender-based differences should be further explored in future studies to explore the interactions between gender, adult depression, and childhood maltreatment, including exploring different types of childhood maltreatment. No differences were found in the effects of problematic mobile phone use on depression and anxiety symptoms among male and female students. Further studies are needed to unravel the inconsistent prevalence of problematic mobile phone use among male and female students and research could explore whether different patterns of mobile phone use affect the onset of depression and anxiety symptoms, and whether there are gender differences in this relationship. The findings of this study suggest the need for a gender-specific approach to effectively help college students improve depression-only symptoms associated with childhood maltreatment.

## Strengths and Limitations

This study had several strengths. First, this study validated the synergistic effects of childhood maltreatment and problematic mobile phone use on depression and anxiety symptoms among college students for the first time, including multiplicative and additive interactions. Second, gender-based differences were observed in the associations. For instance, depression was more common in males and male students with childhood maltreatment were at higher risk of depression-only symptoms. Third, our models evaluated the co-morbidity of depression and anxiety, making the results more realistic.

This study had several limitations that should be acknowledged. First, the cross-sectional nature of the study does not allow causality to be inferred through specific biological and/or socio-psychological factors. Nonetheless, our findings pertaining to the association between childhood maltreatment and problematic mobile phone use with depression and anxiety symptoms were similar to those in previous cohort studies [61, 62]. In addition, Cui et al. found a significant bidirectional relationship between problematic mobile phone use and depression symptoms [63]. Second, all variables were self-reported data; therefore, reporting and recall biases could not be excluded. For instance, there is no complete overlap between self-reported childhood maltreatment and substantiated instances of childhood maltreatment; however, recalled childhood maltreatment tends to be more strongly associated with mental health problems [64]. Thus, self-reported childhood maltreatment data may not inherently be a limitation for being “self-report” but may not have external validity to all circumstances of childhood maltreatment. Third, the participants were recruited from two medical colleges. Although the medical colleges we chose are ranked in the middle of medical universities and colleges in Anhui Province, China (www.cuaa.net), and are a good representation of medical students in Anhui Province, China, the particularities of medical students, such as the high proportion of female students, mean that caution is needed in generalizing the findings of this study to all college students.

Despite these limitations, our study extends previous results regarding the association of childhood maltreatment, problematic mobile phone use, depression, and anxiety by estimating the interaction effects between childhood maltreatment and problematic mobile phone use among college students.

## Conclusion

Our findings suggest that both childhood maltreatment and problematic mobile phone use play a significant role in depression and anxiety symptoms among college students. Importantly, synergistic effects between childhood maltreatment and problematic mobile phone use on depression and anxiety symptoms were observed. Therefore, college students with a history of childhood maltreatment and problematic mobile phone use are at high risk of developing depression and anxiety symptoms, and focusing on childhood maltreatment and problematic mobile phone use could facilitate a reduction in the occurrence of depression and anxiety symptoms in college students. Furthermore, gender-based differences were observed in the association. Male students with a history of childhood maltreatment were at higher risk of depression-only symptoms compared to their female counterparts, indicating the necessity of developing gender-targeted intervention strategies that are useful in preventing and managing depression and anxiety symptoms.

## Electronic supplementary material

Below is the link to the electronic supplementary material.

**Supplementary Material 1: TABLE S1**: Descriptions of socio-demographic characteristics, CM, PMPU and depression and anxiety symptoms characteristics among college student. **TABLE S2**: Outcomes (means, ranges, and correlations) of the subscales of the CTQ-SF among the college students. **TABLE S3**: Full review of multinominal logistic regressions of independent effects between CM and depression and anxiety. **TABLE S4**: Full review of multinominal logistic regressions of independent effects between PMPU and depression and anxiety. **TABLE S5**: Full review of multinominal logistic regressions of multiplicative interaction between CM and PMPU. **TABLE S6**: Additive interactions between CM and PMPU on co-morbidity of depression and anxiety. **TABLE S7**: Quantitative analysis of additive interactions between CM and PMPU on co-morbidity of depression and anxiety. **TABLE S8**: The one-way ANOVA of main and interaction effects of CM and PMPU among college students.

## Data Availability

The datasets generated and/or analyzed during the current study are not publicly available but are available from the corresponding author on reasonable request.
