# Supplementary material for: Gender-based differences in interaction effects between childhood maltreatment and problematic mobile phone use on college students’ depression and anxiety symptoms
Source: BMC Psychiatry. 2023 Apr 25;23:286. doi: 10.1186/s12888-023-04777-x (PMC10127168; doi:10.1186/s12888-023-04777-x)
Supplement: Supplementary file 1 — Supplementary Material 1: TABLE S1: Descriptions of socio-demographic characteristics, CM, PMPU and depression and anxiety symptoms characteristics among college student. TABLE S2: Outcomes (means, ranges, and correlations) of the subscales of the CTQ-SF among the college students. TABLE S3: Full review of multinominal logistic regressions of independent effects between CM and depression and anxiety. TABLE S4: Full review of multinominal logistic regressions of independent effects between PMPU and depression and anxiety. TABLE S5: Full review of multinominal logistic regressions of multiplicative interaction between CM and PMPU. TABLE S6: Additive interactions between CM and PMPU on co-morbidity of depression and anxiety. TABLE S7: Quantitative analysis of additive interactions between CM and PMPU on co-morbidity of depression and anxiety. TABLE S8: The one-way ANOVA of main and interaction effects of CM and PMPU among college students. [file 12888_2023_4777_MOESM1_ESM.docx]

**Supplementary Material**

TABLE S1 Descriptions of socio-demographic characteristics, CM, PMPU and depression and anxiety symptoms characteristics among college student

| Variables | Frequency | Percent (%) |
| --- | --- | --- |
| Grade |  |  |
| freshman | 4211 | 55.2 |
| sophomore | 3412 | 44.8 |
| Gender |  |  |
| male | 1971 | 25.9 |
| female | 5652 | 74.1 |
| Residential area |  |  |
| rural | 6177 | 81.0 |
| urban | 1446 | 19.0 |
| Only child or not |  |  |
| yes | 1636 | 21.5 |
| no | 5987 | 78.5 |
| Perceived family economic status |  |  |
| poor | 2789 | 36.6 |
| moderate | 4583 | 60.1 |
| good | 251 | 3.3 |
| Number of close friends |  |  |
| 0 | 203 | 2.7 |
| 1-2 | 2847 | 37.3 |
| 3-5 | 3350 | 43.9 |
| ≥6 | 1223 | 16.0 |
| CM |  |  |
| yes | 5350 | 70.2 |
| no | 2273 | 29.8 |
| PMPU |  |  |
| yes | 1988 | 26.1 |
| no | 5635 | 73.9 |
| CM*PMPU |  |  |
| no*no | 1841 | 24.2 |
| yes*yes | 1556 | 20.4 |
| no*yes | 432 | 5.7 |
| yes*no | 3794 | 49.8 |
| Depression or anxiety |  |  |
| depression and anxiety | 771 | 10.1 |
| depression only | 750 | 9.8 |
| anxiety only | 779 | 10.2 |
| no depression or anxiety | 5323 | 69.8 |

CM: Childhood maltreatment.

PMPU: Problematic mobile phone use.

TABLE S2 Outcomes (means, ranges, and correlations) of the subscales of the CTQ-SF among the college students

| Variables | Emotional Abuse (EA) | Physical Abuse (PA) | Sexual Abuse (SA) | Emotional Neglect (EN) | Physical Neglect (PN) |
| --- | --- | --- | --- | --- | --- |
| Means | 6.79 | 5.58 | 5.24 | 10.71 | 8.06 |
| Ranges | 5 - 25 | 5 - 25 | 5 - 25 | 5 - 25 | 5 - 25 |
| Childhood maltreatment(%) | 1338(17.6) | 547(7.2) | 857(11.2) | 3907(51.3) | 3623(47.5) |
| *χ*^2^-value | 713.122 | 203.426 | 265.968 | 319.598 | 281.962 |
| *P*-value | <0.001 | <0.001 | <0.001 | <0.001 | <0.001 |

TABLE S3 Full review of multinominal logistic regressions of independent effects between CM and depression and anxiety

| Variables | Depression only^a^ | | |  | Anxiety only^a^ | | |  | Depression and Anxiety^a^ | | |
| --- | --- | --- | --- | --- | --- | --- | --- | --- | --- | --- | --- |
|  | OR | 95% CI | *P*-value |  | OR | 95% CI | *P*-value |  | OR | 95% CI | *P*-value |
| CM |  |  |  |  |  |  |  |  |  |  |  |
| no | 1.00 |  |  |  | 1.00 |  |  |  |  |  |  |
| yes | 2.845 | 2.303-3.516 | <0.001 |  | 1.496 | 1.262-1.774 | <0.001 |  | 4.823 | 3.747-6.208 | <0.001 |
| Gender | 0.786 | 0.659-0.938 | 0.008 |  | 1.013 | 0.845-1.213 | 0.892 |  | 0.852 | 0.713-1.019 | 0.080 |
| Only child or not | 0.750 | 0.622-0.904 | 0.003 |  | 1.015 | 0.837-1.230 | 0.883 |  | 0.891 | 0.733-1.083 | 0.246 |
| Perceived family economic status | 0.718 | 0.619-0.831 | <0.001 |  | 0.922 | 0.799-1.065 | 0.269 |  | 0.709 | 0.612-0.821 | <0.001 |
| Number of close friends | 0.624 | 0.560-0.695 | <0.001 |  | 0.899 | 0.811-0.997 | 0.043 |  | 0.564 | 0.505-0.629 | <0.001 |

^a^: Adjusting for grade, gender, residential area, only child or not, perceived family economic status, number of close friends.

CM: Childhood maltreatment.

TABLE S4 Full review of multinominal logistic regressions of independent effects between PMPU and depression and anxiety

| Variables | Depression only^a^ | | |  | Anxiety only^a^ | | |  | Depression and Anxiety^a^ | | |
| --- | --- | --- | --- | --- | --- | --- | --- | --- | --- | --- | --- |
|  | OR | 95% CI | *P*-value |  | OR | 95% CI | *P*-value |  | OR | 95% CI | *P*-value |
| PMPU |  |  |  |  |  |  |  |  |  |  |  |
| no | 1.00 |  |  |  | 1.00 |  |  |  |  |  |  |
| yes | 2.163 | 1.820-2.571 | <0.001 |  | 4.780 | 4.081-5.598 | <0.001 |  | 8.210 | 6.959-9.685 | <0.001 |
| Gender | 0.722 | 0.606-0.860 | <0.001 |  | 0.983 | 0.818-1.181 | 0.855 |  | 0.786 | 0.652-0.946 | 0.011 |
| Only child or not | 0.776 | 0.645-0.934 | 0.007 |  | 1.039 | 0.853-1.265 | 0.702 |  | 0.929 | 0.759-1.136 | 0.473 |
| Perceived family economic status | 0.695 | 0.600-0.805 | <0.001 |  | 0.908 | 0.785-1.051 | 0.196 |  | 0.689 | 0.592-0.802 | <0.001 |
| Number of close friends | 0.604 | 0.542-0.672 | <0.001 |  | 0.909 | 0.818-1.011 | 0.078 |  | 0.553 | 0.493-0.620 | <0.001 |

^a^: Adjusting for grade, gender, residential area, only child or not, perceived family economic status, number of close friends.

PMPU: Problematic mobile phone use.

TABLE S5 Full review of multinominal logistic regressions of multiplicative interaction between CM and PMPU

| Variables | Depression only^a^ | | |  | Anxiety only^a^ | | |  | Depression and Anxiety^a^ | | |
| --- | --- | --- | --- | --- | --- | --- | --- | --- | --- | --- | --- |
|  | OR | 95% CI | *P*-value |  | OR | 95% CI | *P*-value |  | OR | 95% CI | *P*-value |
| CM*PMPU |  |  |  |  |  |  |  |  |  |  |  |
| no*no | 1.00 |  |  |  | 1.00 |  |  |  | 1.00 |  |  |
| yes*yes | 5.990 | 4.502-7.971 | <0.001 |  | 6.853 | 5.362-8.758 | <0.001 |  | 29.779 | 20.724-42.789 | <0.001 |
| no*yes | 3.081 | 2.028-4.683 | <0.001 |  | 6.361 | 4.695-8.617 | <0.001 |  | 6.700 | 4.120-10.897 | <0.001 |
| yes*no | 3.143 | 2.434-4.059 | <0.001 |  | 1.634 | 1.294-2.065 | <0.001 |  | 3.846 | 2.670-5.541 | <0.001 |
| Gender | 0.790 | 0.662-0.943 | 0.009 |  | 1.025 | 0.852-1.232 | 0.796 |  | 0.862 | 0.714-1.041 | 0.123 |
| Only child or not | 0.752 | 0.623-0.907 | 0.003 |  | 1.025 | 0.841-1.249 | 0.805 |  | 0.900 | 0.733-1.105 | 0.315 |
| Perceived family economic status | 0.717 | 0.619-0.831 | <0.001 |  | 0.919 | 0.794-1.064 | 0.260 |  | 0.714 | 0.612-0.832 | <0.001 |
| Number of close friends | 0.631 | 0.566-0.703 | <0.001 |  | 0.926 | 0.832-1.030 | 0.155 |  | 0.584 | 0.520-0.656 | <0.001 |

^a^: Adjusting for grade, gender, residential area, only child or not, perceived family economic status, number of close friends.

CM: Childhood maltreatment.

PMPU: Problematic mobile phone use.

TABLE S6 Additive interactions between CM and PMPU on co-morbidity of depression and anxiety

| Variables | Total^a^ | | |  | Male^a^ | | |  | Female^a^ | | |
| --- | --- | --- | --- | --- | --- | --- | --- | --- | --- | --- | --- |
|  | OR | 95% CI | *P*-value |  | OR | 95% CI | *P*-value |  | OR | 95% CI | *P*-value |
| CM*PMPU |  |  |  |  |  |  |  |  |  |  |  |
| no*no | 1.00 |  |  |  | 1.00 |  |  |  | 1.00 |  |  |
| yes*yes | 18.822 | 13.044-27.158 | <0.001 |  | 19.866 | 9.004-43.830 | <0.001 |  | 18.302 | 12.132-27.609 | <0.001 |
| no*yes | 4.759 | 2.944-7.691 | <0.001 |  | 4.145 | 1.459-11.781 | 0.008 |  | 4.948 | 2.883-8.494 | <0.001 |
| yes*no | 3.387 | 2.373-4.836 | <0.001 |  | 2.927 | 1.346-6.364 | 0.008 |  | 3.568 | 2.376-5.357 | <0.001 |

^a^: Adjusting for grade, gender, residential area, only child or not, perceived family economic status, number of close friends.

CM: Childhood maltreatment.

PMPU: Problematic mobile phone use.

TABLE S7 Quantitative analysis of additive interactions between CM and PMPU on co-morbidity of depression and anxiety

| Indicator | Total^a^ | | |  | Male^a^ | | |  | Female^a^ | | |
| --- | --- | --- | --- | --- | --- | --- | --- | --- | --- | --- | --- |
|  | Estimate | Lower | Upper |  | Estimate | Lower | Upper |  | Estimate | Lower | Upper |
| RERI | 11.675 | 6.913 | 16.438 |  | 13.793 | 2.542 | 25.045 |  | 10.786 | 5.734 | 15.837 |
| AP | 0.620 | 0.516 | 0.724 |  | 0.694 | 0.518 | 0.871 |  | 0.589 | 0.464 | 0.715 |
| S | 2.900 | 2.106 | 3.993 |  | 3.719 | 1.832 | 7.550 |  | 2.655 | 1.865 | 3.780 |

^a^: Adjusting for grade, gender, residential area, only child or not, perceived family economic status, number of close friends.

CM: Childhood maltreatment.

PMPU: Problematic mobile phone use.

TABLE S8 The one-way ANOVA of main and interaction effects of CM and PMPU among college students

| Variables | Depression^a^ | | |  | Anxiety^a^ | | |
| --- | --- | --- | --- | --- | --- | --- | --- |
|  | F | *P*-value | η2 |  | F | *P*-value | η2 |
| CM | 17.301 | **<0.001** | 0.142 |  | 6.214 | **<0.001** | 0.056 |
| PMPU | 10.527 | **<0.001** | 0.077 |  | 20.720 | **<0.001** | 0.141 |
| CM*PMPU | 1.568 | **<0.001** | 0.203 |  | 1.342 | **<0.001** | 0.178 |
| Gender | 4.378 | 0.036 | 0.001 |  | 5.521 | 0.019 | 0.001 |
| Only child | 20.096 | <0.001 | 0.003 |  | 0.227 | 0.634 | <0.001 |
| Perceived family economic status | 23.104 | <0.001 | 0.004 |  | 1.784 | 0.182 | <0.001 |
| Number of close friends | 102.653 | <0.001 | 0.016 |  | 22.300 | <0.001 | 0.003 |

^a^: Adjusting for grade, gender, residential area, only child or not, perceived family economic status, number of close friends.

CM: Childhood maltreatment.

PMPU: Problematic mobile phone use.
